# Supplementary material for: Vitamin D Modified DSS-Induced Colitis in Mice via STING Signaling Pathway
Source: Biology (Basel). 2025 Jun 18;14(6):715. doi: 10.3390/biology14060715 (PMC12190092; doi:10.3390/biology14060715)
Supplement: Supplementary file 1 [file biology-14-00715-s001.zip › Supplementary Materials S1: 16s-report of gut microbiota/Groups/diff_analysis/Correlation/GBM-GMM.pdf]

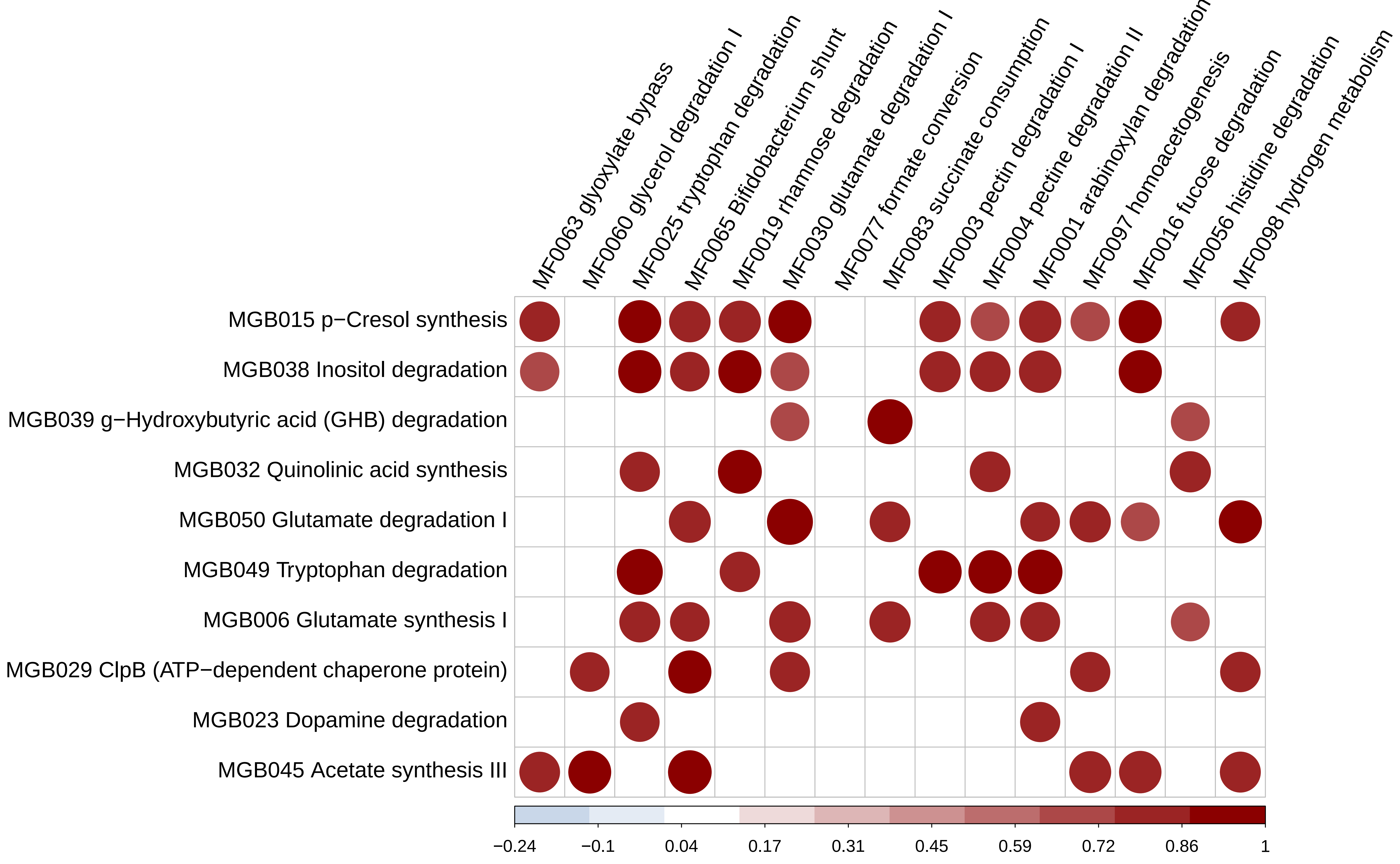
MF0063 glyoxylate bypass
MF0060 glycerol degradation I
MF0025 tryptophan degradation
MF0065 Bifidobacterium shunt
MF0019 rhamnose degradation
MF0030 glutamate degradation I
MF0077 formate conversion
MF0083 succinate consumption
MF0003 pectin degradation I
MF0004 pectine degradation II
MF0001 arabinoxylan degradation
MF0097 homoacetogenesis
MF0016 fucose degradation
MF0056 histidine degradation
MF0098 hydrogen metabolism
MGB015 p−Cresol synthesis
MGB038 Inositol degradation
MGB039 g−Hydroxybutyric acid (GHB) degradation
MGB032 Quinolinic acid synthesis
MGB050 Glutamate degradation I
MGB049 Tryptophan degradation
MGB006 Glutamate synthesis I
MGB029 ClpB (ATP−dependent chaperone protein)
MGB023 Dopamine degradation
MGB045 Acetate synthesis III
−0.24
−0.1
0.04
0.17
0.31
0.45
0.59
0.72
0.86
1
